# A Novel Technique for Pancreatic Stump Closure: Clip Ligation of the Duct and Associated Suturing of Pancreas

**DOI:** 10.7759/cureus.7414

**Published:** 2020-03-25

**Authors:** Michail Papoulas, Elissaios Kontis, Olympia Hadjicosta, Nathaneal Pinsker, Nigel Heaton, Krishna V Menon

**Affiliations:** 1 Institute of Liver Studies, King's College Hospital NHS Foundation Trust, London, GBR; 2 Medical School, Barts and the London School of Medicine and Dentistry, London, GBR

**Keywords:** pancreas, distal pancreatectomy, stump closure, technique, laparoscopic, open

## Abstract

Pancreatic fistula (PF) remains the primary source of morbidity after distal pancreatectomy (DP). There is currently no optimal stump closure technique to reduce PF rates. We present a novel technique for pancreatic stump closure using Clip Ligation of the duct and Associated Suturing of Pancreas (CLASP). Five patients (three females) with a median age of 65 years underwent DP and splenectomy for pancreatic body or tail tumour using the CLASP technique. Four of those operations were done laparoscopically. Only one patient developed grade A PF. No other postoperative complications were noticed. The mean length of stay was 5.4 days. The CLASP technique was applicable in both laparoscopic and open distal pancreatectomy. The key points include mobilisation of the pancreatic body from the retroperitoneum and division of the parenchyma with energy device. The technique of pancreatic stump closure involves the isolation of the pancreatic duct (PD), application of a double ligaclip on the proximal duct, division of the PD and finally suturing of the pancreatic stump. The CLASP technique is an effective and safe alternative technique to the current traditional methods of pancreatic stump closure.

## Introduction

Distal pancreatectomy (DP) was first performed by Billroth in 1884 and was further outlined by Mayo in 1913 [1]. Despite the experience gained since then, the closure of the pancreatic stump is still a considerable clinical problem. The incidence of pancreatic fistula (PF) after DP remains high at 16% to 34% of cases, and it is the primary source of morbidity and life-threatening complications such as intra-abdominal abscess, sepsis and haemorrhage [2]. Multiple risk factors for pancreatic leakage have been identified over the last two decades. The most widely recognised are soft pancreatic parenchyma and small non-dilated duct [3-5] .

Many techniques have been proposed for the management of the pancreatic remnant to reduce the incidence of PF after DP. Stapler closure and handsewn closure of the pancreatic stump are the standard methods described in the literature [2,6-8]. Several other strategies, such as bipolar scissors, fibrin glue sealant, omental plug, falciform ligament patch, saline-linked radiofrequency ablation and pancreaticojejunostomy of the pancreatic stump have also been described [9-13]. However, none of the current techniques of stump closure has proven to be satisfactory for all patients [2,14-16]. 

We present a novel technique for pancreatic stump closure using Clip Ligation of the duct and Associated Suturing of the Pancreas (CLASP). We retrospectively reviewed a prospective database of five patients who underwent DP and splenectomy (DPS) using the CLASP technique by a single surgeon (K.M.). Clinicopathological data and outcomes of the patients were recorded and analyzed. Postoperative complications were classified according to the Clavien-Dindo methodology, and PF was graded according to the International Study Group of PF [17-19].

## Technical report

The initial standard steps that we follow for the DPS include the division of the gastrocolic ligament and short gastric vessels, the mobilisation of the inferior and superior pancreatic borders, and the tunnelling below the pancreatic body/neck, proximally to the lesion. The splenic artery and splenic vein are identified, isolated and divided separately at the level of the planned parenchyma transection. 

**Figure 1:**
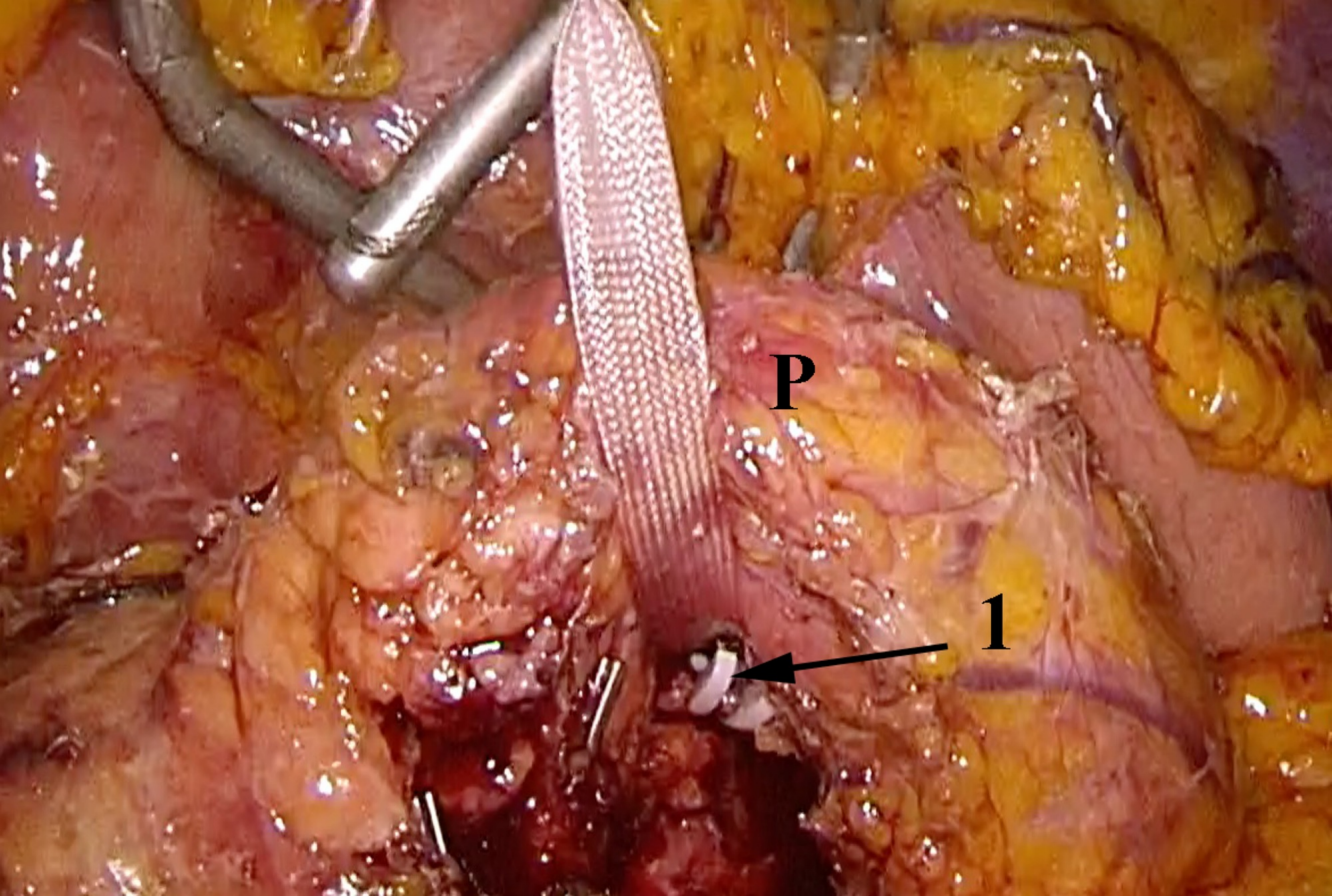
Laparoscopic distal pancreatectomy using the CLASP technique Upward traction of the pancreas (P) with a tape, splenic vessels ligated with hemolock and divided (black arrow). CLASP, Clip Ligation of the duct and Associated Suturing of the Pancreas.

**Figure 2:**
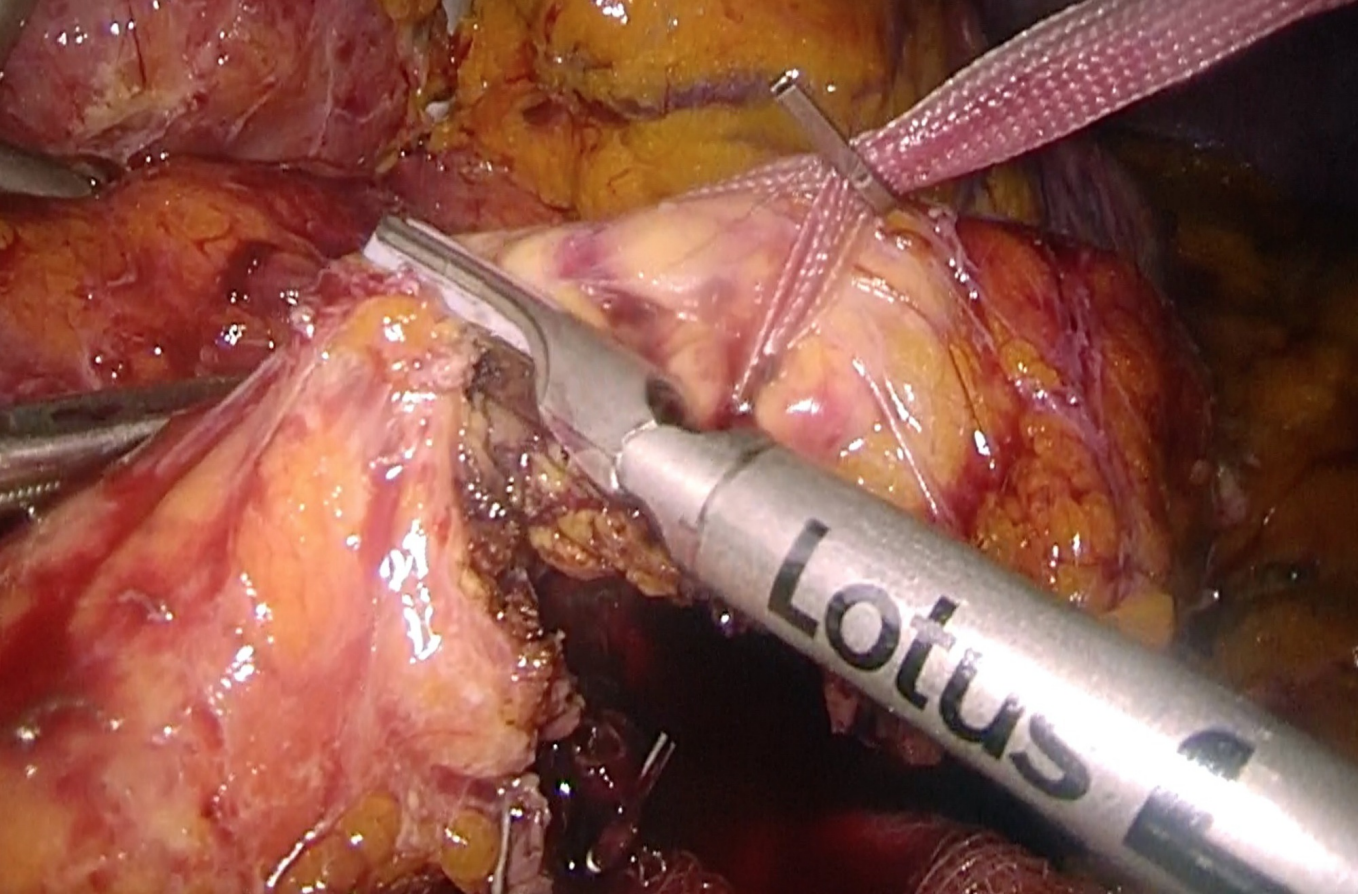
Laparoscopic distal pancreatectomy using the CLASP technique Transection of the pancreatic parenchyma with ultrasonic scissors. CLASP, Clip Ligation of the duct and Associated Suturing of the Pancreas.

**Figure 3:**
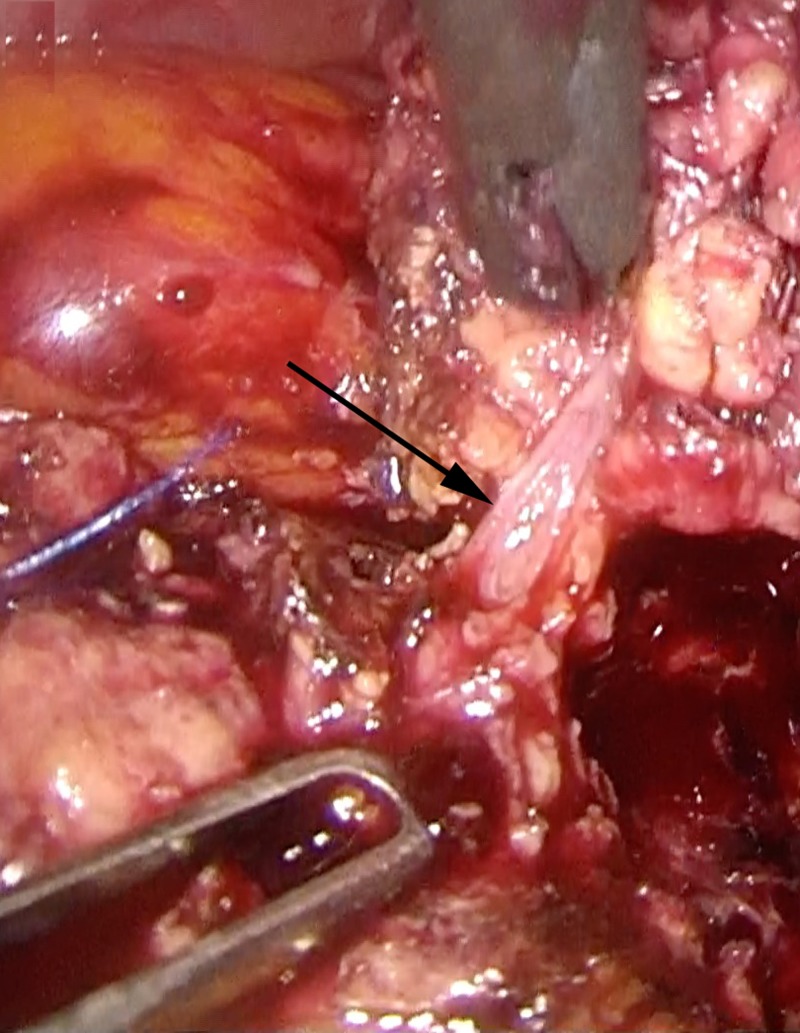
Laparoscopic distal pancreatectomy using the CLASP technique Identification of the main pancreatic duct partially opened with scissors (black arrow). CLASP, Clip Ligation of the duct and Associated Suturing of the Pancreas.

**Figure 4:**
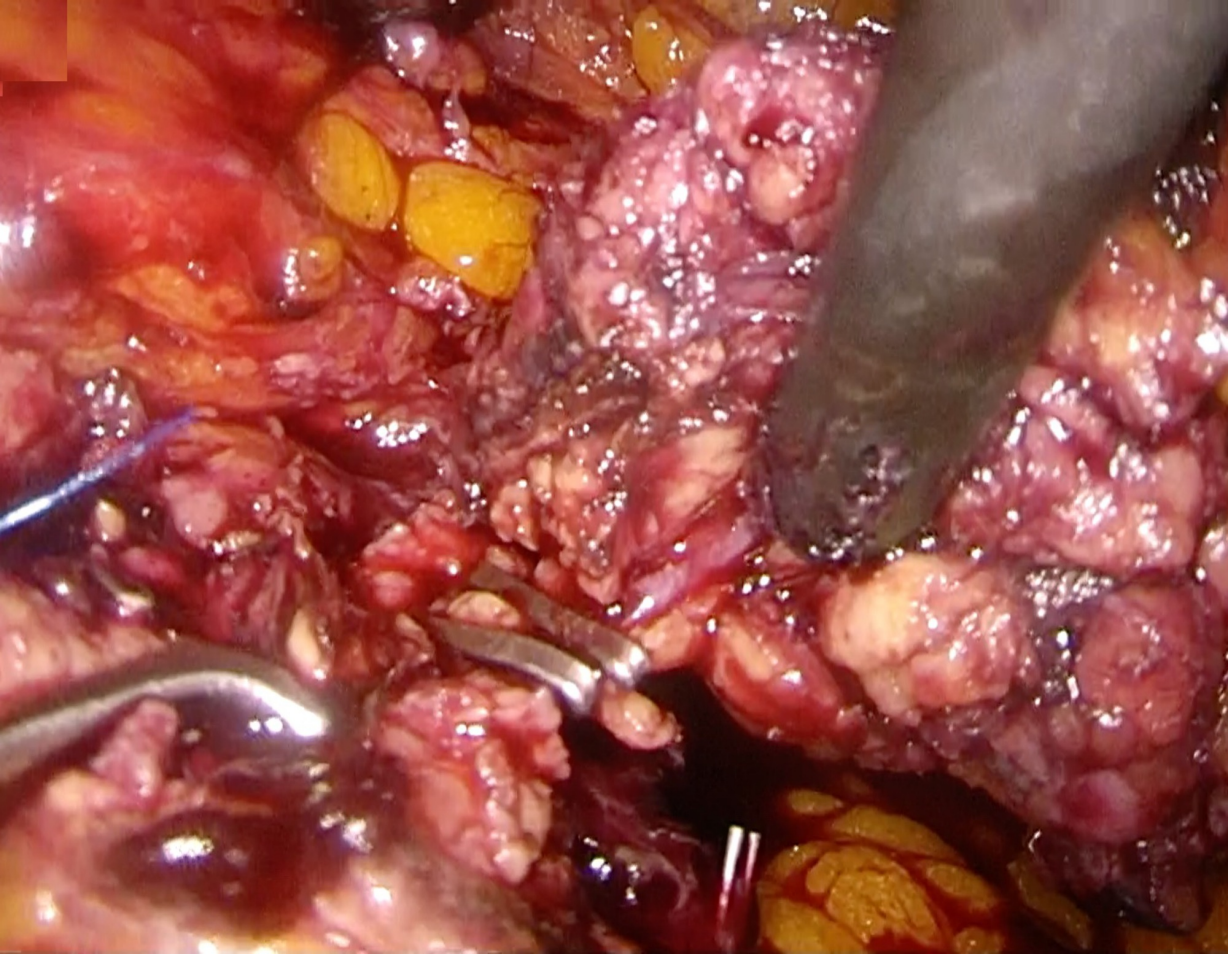
Laparoscopic distal pancreatectomy using the CLASP technique Clip ligation of the main pancreatic duct. CLASP, Clip Ligation of the duct and Associated Suturing of the Pancreas.

**Figure 5:**
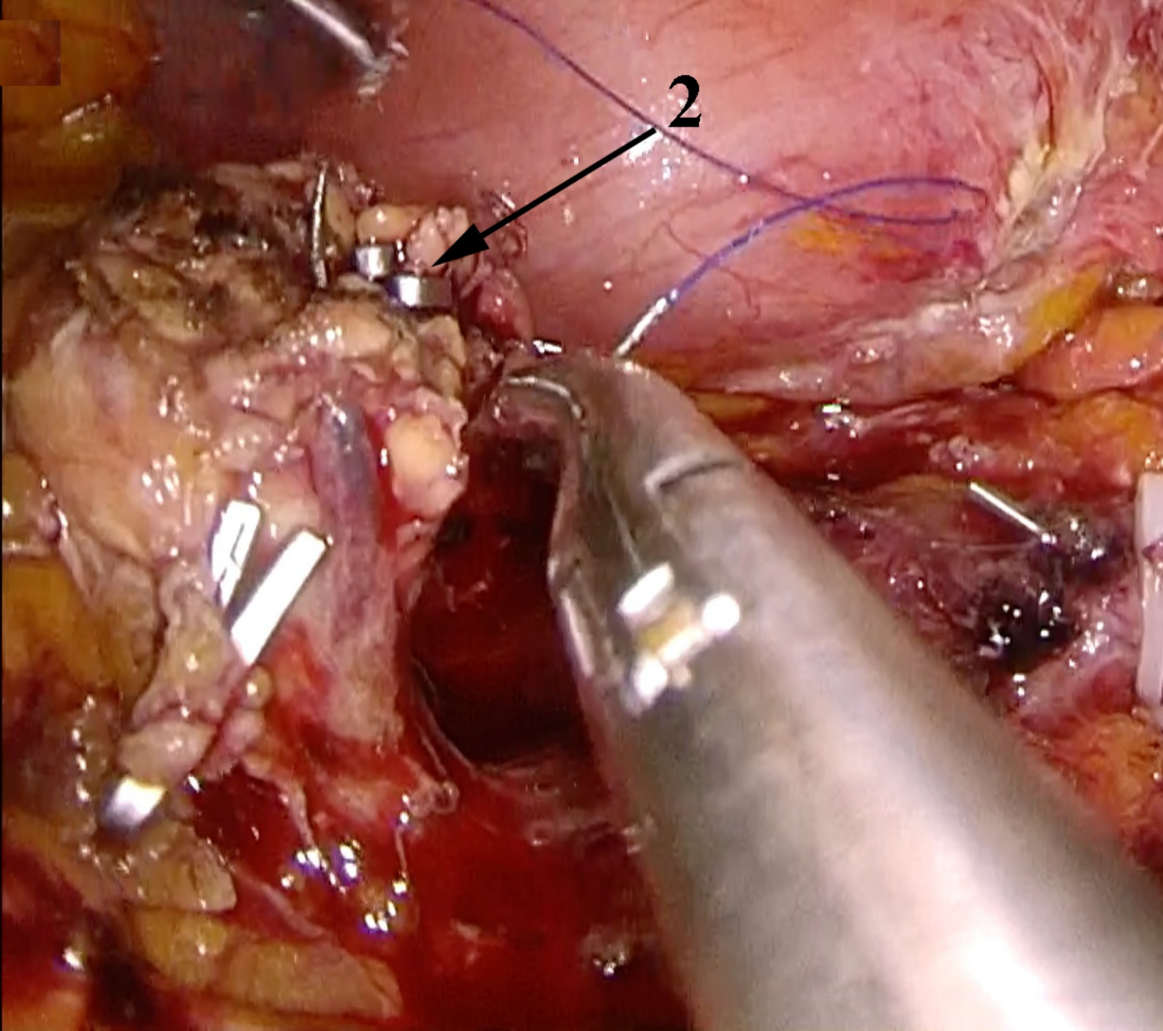
Laparoscopic distal pancreatectomy using the CLASP technique Suturing of the pancreatic stump. Ligated main pancreatic duct pointed with an arrow. CLASP, Clip Ligation of the duct and Associated Suturing of the Pancreas.

**Figure 6:**
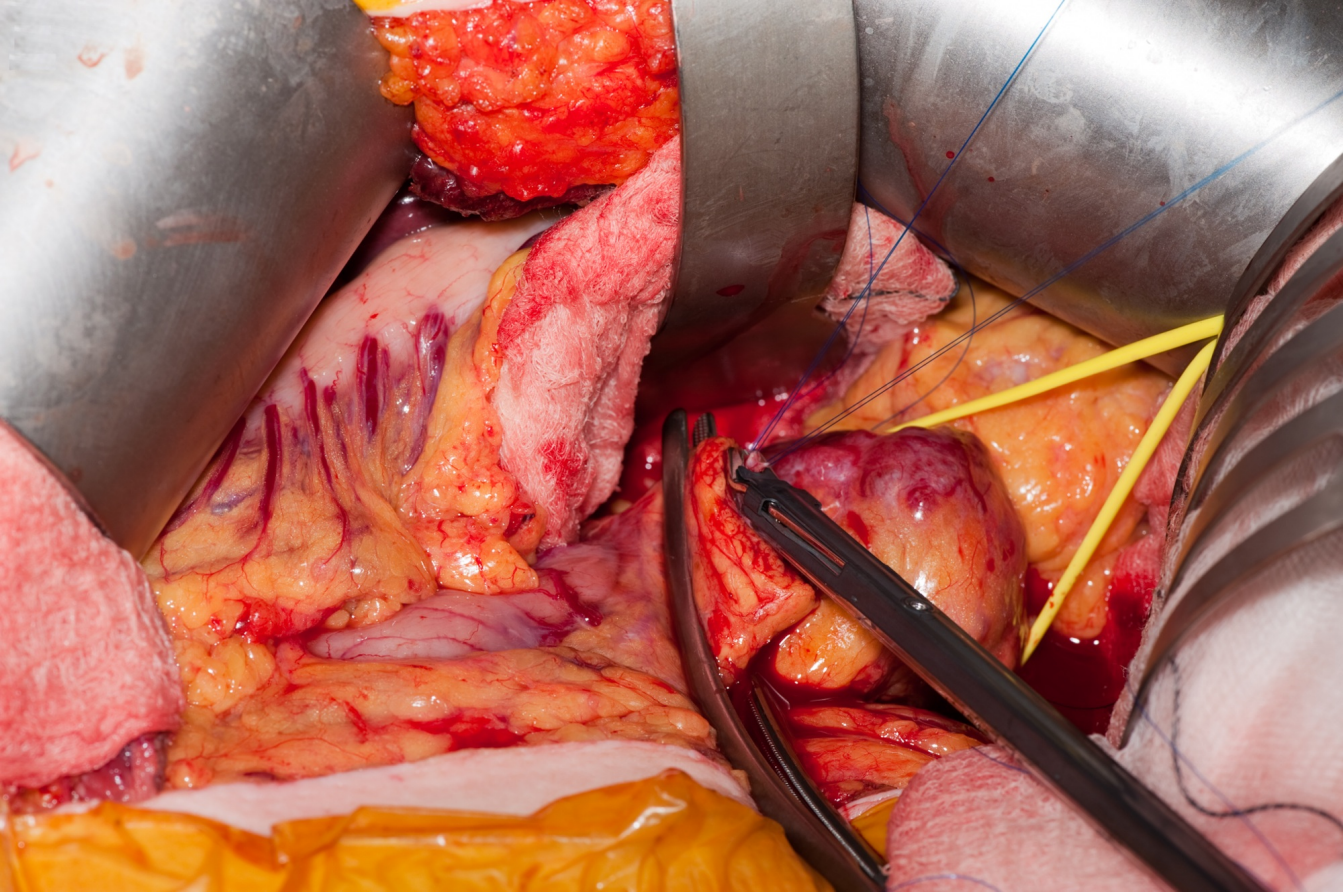
Open central pancreatectomy using the CLASP technique Identification and ligation of the main pancreatic duct with two ligaclips. CLASP, Clip Ligation of the duct and Associated Suturing of the Pancreas.

**Figure 7:**
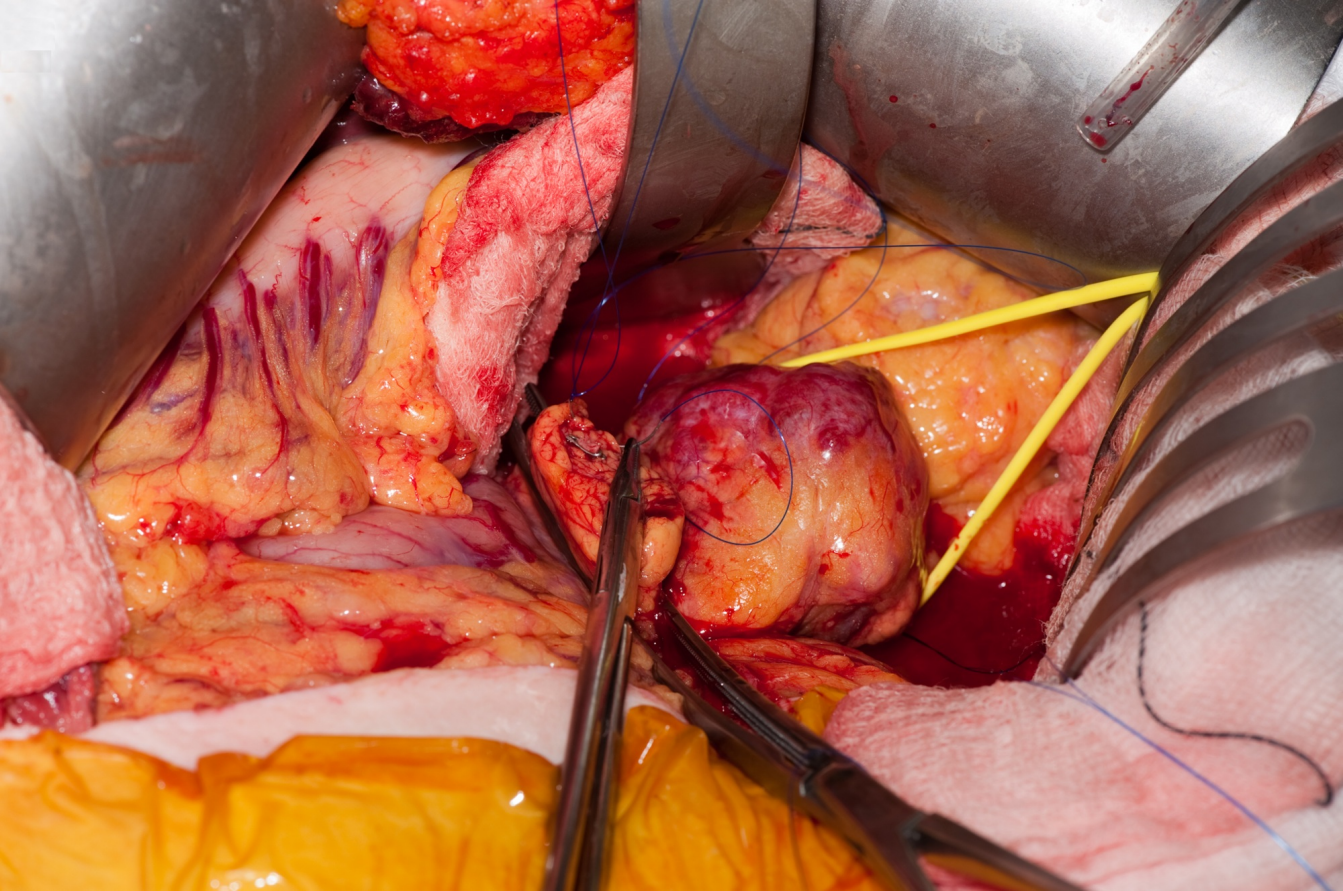
Open central pancreatectomy using the CLASP technique Running suture of the pancreatic stump without going through the main pancreatic duct. CLASP, Clip Ligation of the duct and Associated Suturing of the Pancreas.

**Figure 8:**
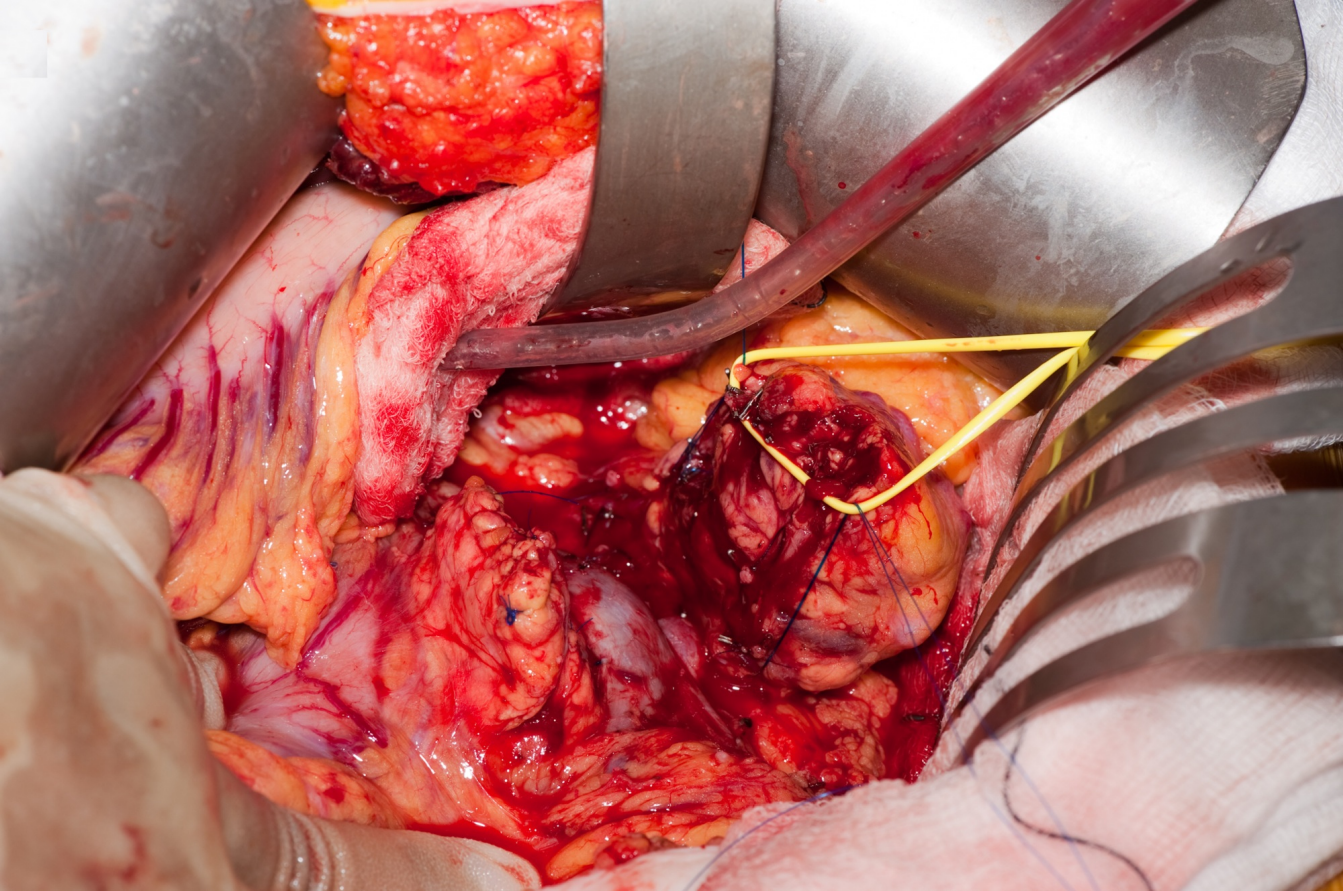
Open central pancreatectomy using the CLASP technique End result of the CLASP technique. CLASP, Clip Ligation of the duct and Associated Suturing of the Pancreas.

The CLASP technique for the closure of the pancreatic stump is applicable for both open and laparoscopic surgery following the same principles and key points (Figures 1-8). The pancreatic parenchyma is mobilised from the retroperitoneal attachments at the level of the transection. The pancreatic parenchyma is divided at the level of the superior mesenteric vein or at the location that is appropriate for the particular pathology to ensure an adequate margin. Careful parenchyma transection should be done using an ultrasonic energy device. The Lotus (BOWA Medical, Ashburton, UK) ultrasonic scalpel (dissecting shears, CV3-400 transducer, DC4-400CD Handpiece) was used in all our cases. Attention should be given when approaching the main pancreatic duct (MPD) within the pancreas, the identification of which can be facilitated by intraoperative ultrasound. Gentle crush clamp using the energy device or an atraumatic clamp can facilitate the identification and isolation of the MPD. The MPD can then be secured by application of two or three ligaclips (Ligaclip 10-M/L, Ethicon Endo-Surgery Inc, Cincinnati, OH) on the proximal MPD and then divided. The final step of the CLASP technique consists of the oversewing the pancreatic stump using a single layer, continuous 4.0 prolene suture (Ethicon, Somerville, NJ) approaching the lips of the pancreatic remnant. Care should be taken not to go through the clipped MPD while suturing. 

 

Overall, five patients underwent DPS using the CLASP technique. Table 1 depicts their clinicopathological characteristics and Table 2 depicts their perioperative outcomes. The mean age of patients was 59.8 years (range, 49-71 years). Four patients underwent laparoscopic DPS and one patient underwent open central pancreatectomy. Small non-dilated pancreatic duct and soft pancreas were present in two and two cases, respectively. The mean operative time was 238 minutes (range, 228-242 minutes). Only one patient developed a biochemical leak with no clinical importance (Clavien-Dindo I). There was no incidence of clinically significant grade B or C PF or any other postoperative complication. The mean length of hospital stay was 5.4 days (range, 5-8 days). Clear resection margins achieved in all patients (R0 resection).

**Table 1:** Clinical and pathological patients' characteristics DPS, distal pancreatectomy and splenectomy; MCN, mucinous cystic neoplasm; IPMN, intraductal papillary mucinous neoplasm, PNET, pancreatic neuroendocrine tumour.

Patient no	Gender	Age, years	Operation	Histology
1	F	49	Lap DPS	MCN, moderate dysplasia
2	F	57	Lap DPS	MCN, mild to moderate dysplasia
3	M	71	Lap DPS	Side branch IPMN, no malignancy
4	M	64	Open central pancreatectomy	Serous cystadenoma
5	F	58	Lap DPS	PNET

**Table 2:** Perioperative outcomes PD, pancreatic duct; OR, operation.

Patient no	Pancreatic paenchyma texture	PD size	OR time (minutes)	Pancreatic fistula	Complications (Clavien-Dindo)	Length of stay (days)
1	Soft	Non-dilated	242	No	Nil	5
2	Hard	Non-dilated	228	No	Nil	4
3	Soft	Dilated	231	No	Nil	5
4	Hard	Dilated	262	No	Nil	8
5	Hard, bulky	Dilated	230	Grade A	Grade I	5

## Discussion

The main effort of all the techniques for the closure of the pancreatic stump is to decrease the incidence of PF. It is well known that the pancreatic leak from the pancreatic parenchyma and small pancreatic duct branches is usually self-controlled and rarely causes clinically relevant PF (grade B or C). On the contrary, leak from the MPD is the primary source of morbidity after DP [20]. There is currently no optimal stump closure technique to reduce the rate of PF, and innovative surgical techniques need to be identified to reduce this adverse outcome [13-15]. 

Our novel technique could be an alternative way of closing the pancreatic stump after DP, comparable to the traditional ones that have been described previously. The focus is on two key points: firstly the division of the pancreatic parenchyma using an energy device that seals the small pancreatic duct branches and secondly the identification and isolation of the pancreatic duct followed by the precise and accurate application of two or three metal clips on the proximal MPD. The pancreatic cut surface is oversewn with a continuous 4.0 prolene suture, closing the lips of the pancreatic remnant. No further adjunct, such as omental plug, falciform ligament patch or fibrin glue sealant, is necessary. We believe that the incidence of leak is reduced when the MPD is identified and directly clipped.

The CLASP technique is applicable to both open and laparoscopic surgery, based on the same principles. It is easily reproducible using an energy device and ligaclips that are routinely used in laparoscopic DPS. We believe that the CLASP technique can be routinely used to close all pancreatic stumps, but also has two specific indications. The first indication is for bulky and thick pancreas where the application of a stapler could crush the parenchyma, increasing the risk of pancreatic leak [8]. The second indication is for lesions located in the proximal body or neck of pancreas where achieving clear macroscopic resection margins could be challenging. In those cases, the distance of the tumour from the plane of transection is limited, even when performing an extended left pancreatectomy, and the application of a stapler could further compromise the resection margin. In that situation, dividing the pancreatic parenchyma using the CLASP technique could provide a safe and clear resection margin. 

The present study has limitations, which are mainly inherent to the small number of patients who underwent DPS using the CLASP technique. Moreover, the results should be validated using large cohorts comparing all the methods of pancreatic stump closure taking into consideration the appearance of the pancreas, the size of the duct and the anatomical and pathological characteristics of the lesion. 

## Conclusions

The CLASP technique appears to be feasible, reproducible and safe alternative technique that can be used for the pancreatic stump closure, compared to the traditional methods of pancreatic stump closure. Particular indications include the presence of bulky pancreas and proximal pancreatic body or neck lesions. Further clinical studies including prospective randomised controlled trials could establish a standardised approach for the pancreatic stump closure.
